# First-Year Findings on Dyslipidaemia Screening in Lithuanian Children: A Focus on Cardiovascular Risk

**DOI:** 10.3390/medicina61040615

**Published:** 2025-03-28

**Authors:** Odeta Kinciniene, Matas Zekonis, Viktoras Sutkus, Ramune Vankeviciene, Juste Parnarauskiene, Zaneta Petrulioniene, Urte Aliosaitiene, Rimante Cerkauskiene

**Affiliations:** Faculty of Medicine, Institute of Clinical Medicine, Vilnius University, M. K. Ciurlionis Street 21, 01513 Vilnius, Lithuania; matas.zekonis@mf.stud.vu.lt (M.Z.); rimante.cerkauskiene@mf.vu.lt (R.C.)

**Keywords:** dyslipidaemia, familial hypercholesterolaemia, risk factor, body composition, physical activity, children

## Abstract

*Background and Objectives*: Cardiovascular diseases are the leading cause of death in Lithuania, with familial hypercholesterolemia being a significant risk factor. This study aimed to evaluate the prevalence of dyslipidaemia among healthy children and the relation with risk factors for familial hypercholesterolaemia. *Materials and Methods*: This study involved 127 children, aged 5 to 10 years, with a focus on the early identification of dyslipidaemia and familial hypercholesterolaemia. The relationship between body composition, dietary habits, physical activity, and dyslipidaemia was researched and statistically assessed. *Results*: Standard lipid profile analysis revealed that approximately one-third of participants had abnormal lipid profiles. Elevated total cholesterol (TC) was found in 19 participants (15%), elevated LDL cholesterol (LDL-C) in 24 participants (18.9%), elevated triglycerides (TGs) in 19 participants (15%), and reduced HDL cholesterol (HDL-C) in 9 participants (7.1%). Risk for familial hypercholesterolaemia was suspected for 12 participants with LDL-C elevated more than 4 mmol/L or family history of FH. While no significant link was found between dyslipidaemia and body composition, low levels of physical activity were associated with increased total cholesterol levels, suggesting a protective role for regular exercise. Dietary habits, including vegetable, sweet, and flour product consumption, did not show a significant association with dyslipidaemia. *Conclusions*: Dyslipidaemia is fairly common among Lithuanian children. Although this study does not show a significant effect of diet or body composition on lipid levels, it links low levels of physical activity to higher triglyceride values. Due to risk factors not always being present in children with dyslipidaemia, it should not be ruled out in apparently healthy children.

## 1. Introduction

Cardiovascular diseases (CVDs) remain the leading cause of death in Lithuania, accounting for 52.5% of all deaths in 2022 and 52.1% in 2023 [1]. Among the contributing factors to premature atherosclerotic cardiovascular disease (ASCVD) is familial hypercholesterolemia (FH), a genetic condition characterised by elevated low-density lipoprotein cholesterol (LDL-C) levels. FH leads to early atherosclerosis, significantly increasing the risk of premature morbidity and mortality. In Lithuania, with a population of approximately 2.85 million, an estimated 14,240 individuals have heterozygous FH (1 in 200), and 18 individuals have homozygous FH (1 in 160,000) [2,3]. Despite its significance, FH remains underdiagnosed and undertreated, as highlighted in the Prague Declaration of the Children’s Screening for CVD in 2022 [4].

Dyslipidaemia has been studied and documented in populations all around the world. Earlier studies of children in Saudi Arabia, Germany, and the USA have shown similar results between the countries. An increase in total cholesterol was observed in about 7–8% of populations, and an increase in LDL-C was observed in 6–7% of populations; a decrease in HDL-C was observed in 8% of German children, 12.8% of Saudi Arabian children, and 13–15% of American children; and an increase in triglycerides was observed in about 12% of German and American and 9.6% of Saudi Arabian paediatric populations [5,6,7,8,9]. A cross-sectional study from the European Atherosclerosis Society, including 663,093 individuals with FH, of whom 11,848 were children and adolescents under 18 years old, detected that clinical signs of FH are very hard to detect in the paediatric population; however, increased availability and use of LDL-C measurements in the first few years of life could help to reduce the current gap between the prevalence and detection of this problem, reducing the risk of organ damage [10].

However, there are populations that report very high dyslipidaemia rates, such as in the Kashmir Valley region, where hypertriglyceridemia was reported in up to 82.6% of the male population aged 5–9 years [11].

Dyslipidaemia, often rooted in childhood, is a major contributor to atherosclerosis in adults [10]. Unhealthy dietary habits, such as the increased consumption of processed and fast foods, combined with sedentary lifestyles, exacerbate lipid imbalances, making early prevention and diagnosis critical [12]. Although dyslipidaemia in adults has been extensively studied, data on its prevalence and associated risk factors in Lithuanian children are lacking.

The Lithuanian adult population has also been studied for dyslipidaemia. The results were shocking, concluding that 9 out of 10 people have dyslipidaemia and 1 out of 10 people have severe dyslipidaemia [12]. Efforts to diagnose dyslipidaemia continue to this day, and new algorithms for screening are being developed [13]. However, Lithuanian children are not being systematically screened for dyslipidaemia, with most cases being diagnosed by opportunistic screening or cascade screening [13].

This study aims to evaluate the prevalence of dyslipidaemia among healthy children aged 5–10 years and those with risk factors for FH. By identifying modifiable and cumulative risk factors, we seek to contribute to early intervention strategies that can improve long-term cardiovascular health outcomes.

## 2. Materials and Methods

Children aged 5–10 years were enrolled into the biomedical study, “Prevalence of Dyslipidaemias in Children”, conducted at Vilnius University Hospital, Santara Clinics. This study aimed to assess the prevalence of lipid disorders and related risk factors among considered-healthy children, focusing on the early identification and management of dyslipidaemia and FH.

Participants were required to meet specific inclusion criteria, including being free from prior diagnoses of dyslipidaemia or other chronic metabolic conditions. A comprehensive questionnaire was administered to gather data on sociodemographic characteristics, medical histories, detailed dietary patterns, physical activity levels, and family histories of cardiovascular diseases.

The questionnaire on dietary patterns, developed by the authors, consisted of 11 items (vegetables, bread, flour dishes, whole grain foods, sweetened milk, sweets, salty snacks, “fast food”, juice, carbonated beverages, sweetened tea), and participants were asked to rate the frequency of intake for each type of food. Possible responses were “several times daily”, “once a day”, “5–6 times per week”, “3–4 times per week”, “twice per week”, “once a week”, “2–3 times per month”, “once a month”, or “less than once a month or never”. The responses on food types were later grouped into 3 categories (vegetables, sweets, and flour products), and responses on intake frequencies were grouped into “everyday”, “non-daily”, and “never” categories.

The questionnaire on physical activity asked participants to evaluate the duration of weekly physical activity in several settings: physical education lessons at school, sports clubs and after school activities, active time with friends after school, and active time during weekends. Participants were classified as having “enough” physical activity if they engaged in daily physical activity or accumulated at least 7 h per week, while those who did less than this were classified as “not enough”.

Anthropometric and body composition measurements were obtained using the advanced bioimpedance analyser TANITA MC-780MA-N, which provided detailed data on weight, fat mass (both in kilogrammes and percentage), trunk fat content (kg and %), muscle mass (kg), and body mass index (BMI). Standardised protocols ensured consistent and accurate measurements [14].

Additionally, fasting blood samples were collected to analyse lipid profiles, including total cholesterol, low-density lipoprotein cholesterol (LDL-C), high-density lipoprotein cholesterol (HDL-C), and triglycerides (TGs). The risk of FH was assessed using criteria outlined by the US National Institutes of Health and European Atherosclerosis Society guidelines, considering both clinical parameters and family history [2].

In cases where FH was suspected, participants underwent genetic testing using next-generation sequencing (NGS). This analysis targeted mutations in the LDLR, APOB, PCSK9, and LDLRAP1 genes, which are known to contribute to FH. The genetic testing component added a crucial layer to this study, enabling the identification of hereditary lipid disorders and enhancing the precision of risk assessment.

The study protocol was approved by the Vilnius Regional Biomedical Research Ethics Committee of Lithuania (No. 2023/1-1497-955). Written informed consent to participate in this research was obtained from their parents or legal guardians.

All statistical analyses were conducted using IBM SPSS Statistics (version 29.0.0). For comparisons between two groups of quantitative variables, Student’s *t*-test or the nonparametric Mann–Whitney U test was employed, depending on the data distribution. For comparisons involving more than two groups, one-way analysis of variance (ANOVA) or the nonparametric Kruskal–Wallis test was used as per the appropriateness of the data. The normality of the data was assessed using the Shapiro–Wilk test. Categorical variables were compared using Pearson’s chi-square test or Fisher’s exact test, as required. A *p*-value less than 0.05 was deemed statistically significant

## 3. Results

### 3.1. Participant Characteristics

A total of 127 children, aged 5 to 10 years, participated in this study, including 71 females (55.9%) and 56 (44.1%) males. The average age of the participants was 7.37 ± 1.53 years. The mean age for girls was similar to the mean age for boys (7.38 years (±1.53) vs. 7.35 years (±1.52)).

### 3.2. Lipid Profiles

Standard lipid profiles analysis revealed that approximately one-third of participants had abnormal lipid profiles. Elevated TC was found in 19 participants (15%), elevated LDL-C in 24 participants (18.9%), elevated TGs in 19 participants (15%), and low HDL-C in 9 participants (7.1%).

The mean TC level in all the groups of participants was 4.41 mmol/L (±1.14), while the mean LDL-C value was 2.76 mmol/L (±1.02), the mean HDL-C value was 1.55 mmol/L (±0.38), and the mean TG value was 0.78 mmol/L (±0.46). The mean TC, LDL-C, HDL-C, and TG values by gender are presented in Table 1. Boys had slightly higher TC and HDL-C levels, while girls showed higher TG levels, which could reflect some potential gender differences in lipid metabolism.

The median LDL-C levels were 2.64 mmol/L (2.15–3.05) for girls and 2.6 mmol/L (2.16–2.9) for boys, showing no significant difference (*p* = 0.95). Categorically, 33.9% of children had normal LDL-C levels, while 18.9% had high and 47.2% had low LDL-C levels. Among boys, 16.1% had high LDL-C levels compared to 21.1% of girls.

Moreover, the risk for FH was suspected for 12 participants with LDL-C elevated more than 4 mmol/L or family history of FH. To further evaluate this risk, genetic testing was performed on a subset of participants. In total, 11 children with identified dyslipidaemia or a family history were screened for FH, revealing heterozygous mutations in the *LDLR* gene in three cases and the *APOB* gene in one case.

### 3.3. Body Composition Analysis

The median weight was similar between boys and girls, namely weights of 25.1 kg (21.5–32.4) and 25.1 kg (20.8–30.8), respectively, showing no statistically significant difference (*p* = 0.76). In terms of fat mass, boys had a median fat mass of 4.6 kg (3.7–7.2), while girls had a slightly higher median fat mass of 5.5 kg (4.2–7.1), though the difference was not significant (*p* = 0.079).

**Figure 1:**
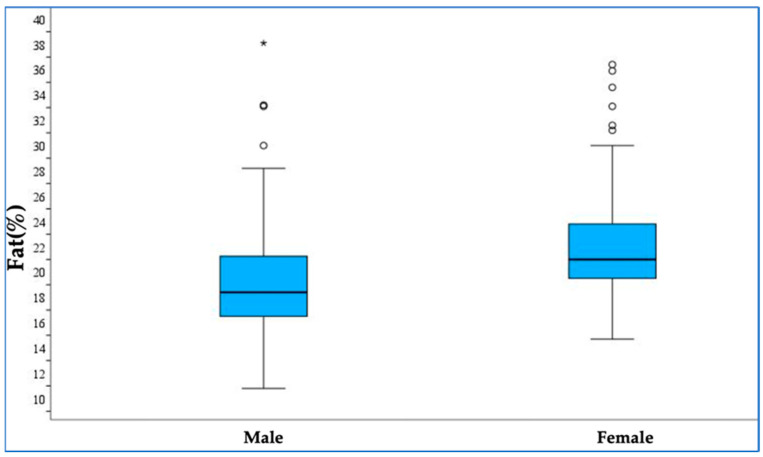
Comparison of body fat mass percentages in boys and girls. Asterisk (*)—marks extreme outliers.

When evaluating children with increased fat mass, 15 boys (29.4%) and 10 girls (15.4%) fell into this category, indicating a trend toward a difference without statistical significance (*p* = 0.068). Conversely, fat mass within the normal range was significantly different between genders: boys had a median fat mass percentage of 18.4% (16.5–21.3), while girls had a higher percentage of 21.0% (19.5–24.2) (*p* < 0.001), which is shown in Figure 1. The trunk fat content also differed between boys and girls. Boys had a median trunk fat content of 1.8 kg (1.5–2.8), compared to 2.2 kg (1.7–3.0) in girls, with the difference being statistically significant (*p* = 0.035). Similarly, when trunk fat was analysed as a percentage, boys had a median of 12.8% (10.8–15.9), while girls had a significantly higher percentage of 15.5% (13.3–18.6) (*p* = 0.001). Other body composition parameters such as muscle mass, BMI, and phase angle were evaluated but did not show significant differences between genders.

Additionally, among the 127 participants, 91 had a normal weight, 7 were underweight, and 29 (22.8%) had a BMI exceeding the 85th percentile. These findings underscore the importance of the early assessment of body composition to understand potential gender-related differences and risk factors for future health outcomes.

After the statistical assessment, we could not find a significant correlation between body composition and dyslipidaemia in our study group. The results are shown in Table 2.

### 3.4. Dietary Habits

The dietary habits of the study participants were evaluated by measuring their frequency of consumption for vegetables, sweets, and flour products. Although the questionnaire covered all the major food groups, only selected groups were included in the statistical analysis. In total, 117 questionnaires were completed by the participants. The results are presented in Table 3.

We found no significant correlation between dietary habits and dyslipidaemia (Table 4).

### 3.5. Physical Activity

A boxplot was created to visually represent the distribution of total cholesterol levels in relation to physical activity time and divided into two groups: those meeting the recommended activity levels (“enough”—at least 7 h/week) and those not meeting them (“not enough”—less than 7 h/week).

**Figure 2:**
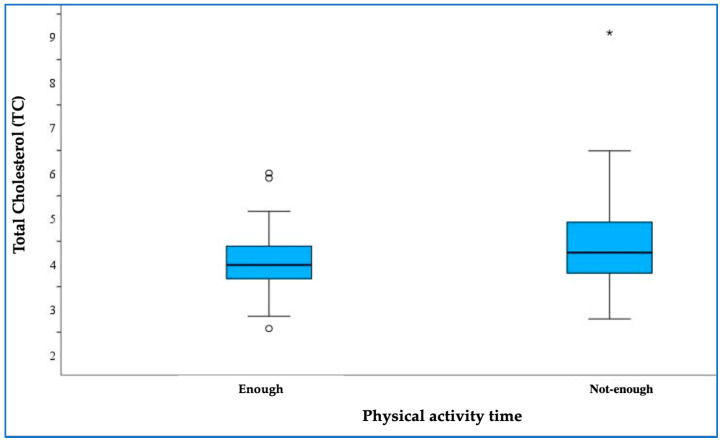
Comparison of TC levels between groups with “enough” and “not enough” physical activity. Asterisk (*)—marks extreme outliers.

The boxplot showed a notable difference in total cholesterol levels between the two groups, with participants classified as ‘not enough’ having higher total cholesterol levels compared to those classified as ‘enough’. The Mann–Whitney test confirmed this difference, yielding a *p*-value of 0.035, indicating statistical significance (Figure 2).

## 4. Discussion

After performing an analysis of the first-year findings provided by our study, we were surprised by the high prevalence of dyslipidaemia among healthy children. However, compared to almost all adults, their dyslipidaemia rates were less prevalent, with about one-third of the children having abnormal lipid test results [12]. The results of this study provide a greater understanding of how various risk factors—body composition, diet, and physical activity—may have an influence on dyslipidaemia.

Despite our detailed analysis into how changes in body composition might affect lipid profiles in children, we could not find any strong links between dyslipidaemia and body composition measurements in the group we studied. This lack of connection could be due to several factors, such as the small sample size, the unique demographic characteristics of the participants, and possible confounding factors like dietary habits, physical activity levels, and genetic tendencies. Additionally, the complex nature of lipid metabolism and its numerous factors could contribute to these results, suggesting that body composition might not fully explain the differences in lipid profiles among children with dyslipidaemia.

We also observed a slightly higher fat mass in girls compared to boys. This could be explained by physiological changes in total body fat. Earlier studies of children have shown that children have a similar mass of body fat before the age of 7, after which fat mass remains steady for boys but starts increasing for girls [14].

In our study, we also examined the potential relationship between some dietary habits and dyslipidaemia. Vegetables, due to the soluble and insoluble fibre they contain, are associated with maintaining normal blood cholesterol levels [7,15]. In our study, we aimed to assess whether the products most eaten by Lithuanian children aged 5–10, such as sweets (candy, gummy bears, chocolate, ice cream, etc.) and flour products, which are mostly refined grains (e.g., pancakes, pasta, dumplings, pastries, etc.), have an impact on changes in cholesterol levels. We did not find a statistically significant difference between the frequency of the consumption of vegetables, sweets, or flour products and dyslipidaemia. Diet is a known risk factor for dyslipidaemia [6,7,15]; therefore, we may only conclude that our measured dietary habits may not be as influential as some others like sugar-sweetened beverages, fruit juice, or food which contains saturated fat or trans-fat.

Our analysis revealed a significant correlation between physical activity and total cholesterol levels, indicating that insufficient physical activity was associated with elevated total cholesterol among participants. This finding suggests that regular exercise may play a crucial role in managing cholesterol levels and serve as a protective factor against dyslipidaemia. Such results highlight the importance of promoting physical activity as an effective intervention to improve lipid profiles and reduce the risk of dyslipidaemia in paediatric populations. However, no significant relationships were observed between physical activity and other lipid profiles, LDL cholesterol, HDL cholesterol, or TGs.

Another study of Portuguese children also evaluated the effect of diet, obesity, and physical activity on lipid levels [16], with children who were obese having worse levels for all lipids compared to non-obese children, and a diet rich in fat, salt, and sugar with fewer vegetables and fruits resulting in more abnormal lipid levels. The study results also showed that an increase in physical activity resulted in a decrease in TG levels. These results differ from our study, which showed only a link between physical activity and TC levels. Possible explanations for these differences are that the Portuguese study included a larger proportion of obese children and a different focus on dietary preferences.

A recent study, based on Familial Hypercholesterolemia Studies Collaboration (FHSC) data, has shown that cardiovascular risk factors were not common in children and adolescents, concluding that the detection of hypercholesterolemia in children relies mostly on laboratory testing [10].

The Lithuanian adult population has already been evaluated in several studies [12,17]. One study of lipid profile evaluation revealed that 89.7% of the studied population had dyslipidaemia, with adults having higher mean TC (6.08 ± 1.21 mmol/L), LDL-C (3.87 ± 1.08 mmol/L), and TG values (1.59 ± 1.16 mmol/L) and similar HDL-C levels (1.54 ± 0.46 mmol/L) compared to children from our study [17]. During another study, risk factors of the Lithuanian adult population were studied. It was shown that there are significantly higher rates of obesity, high body mass index (>30), unbalanced diet, and low physical activity in people with dyslipidaemia compared to people without dyslipidaemia [12]. Such a difference may be partially explained by the tendency of the lipid profile to worsen with age [18] and by different selection methods in the second adult study [12], focusing on severe dyslipidaemia and including subjects with secondary illnesses, whereas we excluded those with suspected metabolic disorders.

When evaluating risk factors, studies on adult populations have shown significantly higher rates of obesity, a high body mass index (>30), unbalanced diet, and low physical activity in people with dyslipidaemia compared to those without. Such differences may be partially explained by the tendency of lipid profiles to worsen with age [13] and by different selection methods, with adult studies focusing on severe dyslipidaemia and including subjects with secondary illnesses, whereas we excluded those with suspected metabolic disorders.

Our multifaceted approach ensured a comprehensive evaluation of lipid disorders and related risk factors, providing valuable insights into the prevalence and potential causes of dyslipidaemia in Lithuanian children. The results obtained from our first-year study will significantly motivate us to continue our research, refine the data, and look for new signs that may lead us to diagnose dyslipidaemia earlier and possibly even decrease cardiovascular morbidity and mortality in adults.

There are, however, some limitations to this study that must be noted. Only a limited number of participants, those with the highest suspicion, underwent genetic screening, which may have resulted in some undiagnosed cases. The dietary habits questionnaire included a limited number of items, and other factors that were not considered may play a greater role in dyslipidaemia. There were also 10 participants in this study who did not complete the dietary habits questionnaire, which may have affected the final evaluation of the dietary effect on the lipid profile.

## 5. Conclusions

Dyslipidaemia is fairly common among Lithuanian children. Although this study does not show a significant effect of diet or body composition on lipid levels, it shows that low levels of physical activity are linked to higher triglyceride values. Due to risk factors not always being present in children with dyslipidaemia, it should not be ruled out in apparently healthy children.

## Figures and Tables

**Table 1:** Lipid panel mean values for the study group by gender.

Lipid Profile	Boys (*n* = 56)	Girls (*n* = 71)
TC	4.5 mmol/L (±1.20)	4.34 mmol/L (±1.09)
LDL-C	2.8 mmol/L (±1.14)	2.75 mmol/L (±0.93)
HDL-C	1.6 mmol/L (±0.39)	1.51 mmol/L (±0.37)
TGs	0.73 mmol/L (±0.48)	0.82 mmol/L (±0.44)

**Table 2:** Correlations between dyslipidaemia and body composition.

Variable	Total CL (r, *p*)	LDL-C (r, *p*)
Weight (kg)	−0.039 (*p* = 0.674)	−0.005 (*p* = 0.960)
BMI	0.013 (*p* = 0.891)	0.055 (*p* = 0.547)
Fat mass (%)	−0.130 (*p* = 0.165)	−0.110 (*p* = 0.240)
Trunk fat (%)	−0.013 (*p* = 0.228)	−0.093 (*p* = 0.323)
Muscle mass (kg)	0.029 (*p* = 0.758)	0.059 (*p* = 0.529)

**Table 3:** Dietary intake of participants by gender.

Food Type	Consumption Frequency	Total (n = 117)	Boys	Girls
	Everyday	79 (67.5%)	34 (65.4%)	45 (69.2%)
Vegetables	Non-daily eating	38 (32.5%)	18 (34.6%)	20 (30.8%)
	Never	0 (0%)	0 (0%)	0 (0%)
	Everyday	65 (55.6%)	29 (55.8%)	36 (55.4%)
Sweets	Non-daily eating	51 (43.6%)	22 (42.3%)	29 (44.6%)
	Never	1 (0.9%)	1 (1.9%)	0 (0%)
	Everyday	50 (42.7%)	23 (44.2%)	27 (41.5%)
Flour products (n%)	Non-daily eating	63 (53.8%)	27 (51.9%)	36 (55.4%)
	Never	4 (3.4%)	2 (3.8%)	2 (3.1%)

**Table 4:** Correlations between lipid profile and dietary habits.

Variable	TC (r, *p*)	LDL-C (r, *p*)
Vegetables	−0.120 (*p* = 0.210	−0.090 (*p* = 0.300)
Sweets	−0.050 (*p* = 0.610)	−0.020 (*p* = 0.790)
Flour products	0.020 (*p* = 0.850)	0.030 (*p* = 0.700)

## Data Availability

The original contributions presented in the study are included in the article, further inquiries can be directed to the corresponding authors.

## Abbreviations

ANOVA	analysis of variance
ASCVD	atherosclerotic cardiovascular disease
BMI	body mass index
CVDs	cardiovascular diseases
FH	familial hypercholesterolemia
FHSC	Familial Hypercholesterolemia Studies Collaboration
HDL-C	high-density lipoprotein cholesterol
LDL-C	low-density lipoprotein cholesterol
NGS	next-generation sequencing
TC	total cholesterol
TG	triglycerides
